# The Time Course of Pulmonary Function Tests in COPD Patients with Different Levels of Blood Eosinophils

**DOI:** 10.1155/2016/4547953

**Published:** 2016-10-16

**Authors:** Paola Rogliani, Ermanno Puxeddu, Chiara Ciaprini, Josuel Ora, Angelo Onorato, Gabriella Pezzuto, Luigino Calzetta, Mario Cazzola

**Affiliations:** Department of Systems Medicine, University of Rome Tor Vergata, Chair of Respiratory Medicine, Rome, Italy

## Abstract

Only very few studies have investigated the influence of eosinophils on the functional progression of COPD. We aimed at retrospectively analyzing the trend of pulmonary function tests over time in patients with COPD according to two baseline blood eosinophil cell count strata (<2% [EOS−] and ≥2% [EOS+]). We used the last 9-year data present in the database of our outpatient clinic and selected only those who had two blood counts that would guarantee the stability of the value of eosinophils and serial spirometry for 4 consecutive years. The analysis of the time course of the spirometric variables analysed showed differences in FEV1 and FVC decline between the subjects of the EOS− group and those of the EOS+ group. The integrated evaluation of our results suggests that the different level of blood eosinophils in the two groups may have influenced independently the time course of the pulmonary function tests and identify two subgroups of subjects with specific disease characteristics: the hyperinflator and the rapid decliner, respectively.

## 1. Introduction

The heterogeneity of COPD events stimulates great interest among clinicians and researchers: in fact, many aspects of the processes involved in clinical expression and progression of COPD remain to be clarified. The role of eosinophils is between factors that should be investigated, in light of their pathogenetic importance in other airways obstructive syndromes, such as bronchial asthma.

Numerous studies have examined the influence of eosinophils in the inflammatory process of COPD but with conflicting results [1]. Although neutrophilic inflammation is commonly reported, a sputum eosinophilia is present in COPD compared with healthy controls and asymptomatic smokers [2, 3]. Furthermore, sputum eosinophilia has been linked with bronchial hyperreactivity in COPD patients [4]. It was also found that during an acute exacerbation, the number of eosinophils in the airways increases 30-fold and that of neutrophils only 3-fold compared with stable COPD [5]. Nevertheless, other authors found no significant differences in the level of eosinophils-related chemokines in elderly patients with COPD with or without atopic symptoms and concluded that the pattern of “allergic inflammation” that is mediated by eosinophils does not play a determining role in the pathogenesis of COPD [6].

Anyway, there is some agreement on the direct relationship between the increase of eosinophils in induced sputum and/or in blood of patients suffering from COPD and a positive response to treatment with corticosteroids [3, 7–11] or to the reversibility test with a short-acting bronchodilator [12, 13].

Inexplicably, only a small number of studies have investigated the influence of eosinophils on the functional progress of COPD, for example, assessing the association between the rapid decline in lung function and some eosinophil related cytokines [14] or circulating eosinophils [15].

Therefore, it is becoming increasingly important to understand the clinical implication that eosinophils may have in COPD and, specifically, to investigate the influence of blood eosinophil count, which is an easily measurable marker, on the progression of COPD. For this reason, we retrospectively analysed the time course of pulmonary function tests in patients with COPD according to two baseline blood eosinophil cell count strata (<2% and ≥2%). This cut-off was chosen in accordance with former studies present in the literature showing that it may identify patients who would benefit from corticosteroids [10, 11]. In any case, in a nationally representative dataset of the US population, a blood eosinophil cell count >2% was prevalent in subjects with COPD (70.7% of participants, estimated 12.8 million of population) [16].

## 2. Material and Methods

We conducted a retrospective observational study using the last 9-year data present in the electronic database of our outpatient clinic at the University Hospital of Tor Vergata, Rome, Italy. This study was approved by our Ethical Committee.

We selected only those patients suffering from COPD who had at least two blood counts within six months that would guarantee the stability of the value of eosinophils over time and serial spirometry for 4 consecutive years. Information on their anthropometric characteristics such as gender, age, weight, body mass index (BMI), smoking status (current smoker, ex-smoker, and pack/years), history of atopy, and prescribed therapies for the respiratory system (long-acting *β*_2_ agonists [LABA]; long-acting muscarinic antagonists [LAMA]; inhaled corticosteroids (ICS); and oxygen) were collected. We have also collected the values of leading spirometry parameters: forced expiratory volume in 1 second (FEV_1_), forced vital capacity (FVC), residual volume (RV), and total lung capacity (TLC), expressed both in absolute terms and as a percentage of the predicted value.

We excluded all patients who, while presenting anamnestic (smoking), clinical (chronic productive cough), and functional (limited bronchial reversibility) characteristics of COPD, had a confounding pulmonary comorbidity such as a lung cancer, an interstitial lung disease, or a possible overlap with asthma.

### 2.1. Statistical Analysis

The difference between the baseline characteristics of the EOS+ and EOS− patients have been assessed by *t*-test or by evaluation of the extent to which the confidence intervals overlapped. Spirometric variables have been normalized for the baseline values. Data have been expressed as the Δ change from baseline values in order to carry out the quantitative time course analysis of pulmonary function tests. The time course of pulmonary function test variables was assessed by applying the following linear regression equation: *Y* = *Y*_intercept + slope*∗X*, where *Y* represents the normalized spirometric variables and slope indicates the average changes of spirometric variables over one year.

The relationship between the time and spirometric variables has been tested by using *P* value for the null hypothesis that the overall slope was zero. *P* value has been calculated from *F*-test and addressed the question of the probability that randomly selected points would result in regression lines as far from horizontal than those observed if there was no linear relationship between *X* and *Y* overall, or, equivalently, the probability that randomly selected points would result in *R* ^2^ values as high as those observed.

Values are presented as mean ± standard error of the mean (SEM) and the graphical representation of linear regressions reports the 95% confidence bands of the best-fit line. The statistical significance was defined as *P* < 0.05, and all data analyses were performed by using the computer software GraphPad Prism version 5.00 for Mac (CA, USA) and OpenEpi (Dean AG, Sullivan KM, Soe MM; Open Source Epidemiologic Statistics for Public Health).

## 3. Results

### 3.1. Characteristics of the Study Population

Three hundred seventy-eight folders of COPD patients that performed spirometry between January 2009 and December 2011 were analysed of which 157 were ruled out because they did not have a repetition of the measurement of eosinophils, 102 patients were excluded because the eosinophils were not stable over the time, and 84 were excluded because they lacked the repeated spirometry.

In total, 35 patients were included in the study. Twelve had an eosinophil count <2% (EOS−) and twenty-three an eosinophil count ≥2% (EOS+). The demographic characteristics of the study population are listed in Table 1. None of the patients had a history of atopy.

The blood eosinophil count was significantly higher in EOS+ compared with EOS− group (3.6 ± 0.2% versus 1.2 ± 0.1%, resp.; *P* < 0.01), whereas the number of cigarettes smoked by EOS+ patients was significantly lower than that of EOS− patients (mean pack-year: 34 ± 4 versus 61 ± 10, resp.; *P* < 0.05).

The distribution of gender, age, and smoking habit was homogeneous between the two groups of patients, although there was a signal for higher BMI in EOS− patients than in EOS+ patients (29.9 ± 2.0 versus 26.6 ± 1.1 kg/m^2^, resp.; *P* = 0.132).

Fifty percent of EOS− patients were on home oxygen therapy compared to nine percent of EOS+ patients (*P* < 0.05). The use of ICSs was greater in EOS+ patients (EOS+: 74% versus EOS−: 58%) and the use of LAMAs was greater in EOS− patients (EOS+: 70% versus EOS−: 83%).

### 3.2. Time Course of the Spirometric Variables in the Two Groups

The analysis of the time course of the spirometric variables analysed showed some differences between the subjects of the EOS− group and those of the EOS+ group (Table 2).

**Figure 1:**
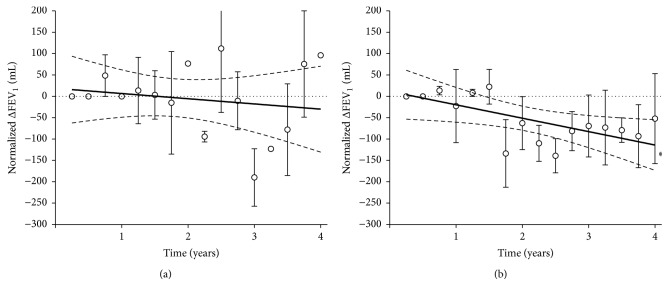
Normalized change from baseline in FEV_1_ in EOS− (a) and EOS+ (b) COPD patients; ^*∗*^slope significantly different from zero (*P* < 0.05). Data points expressed as mean ± SEM and the graphical representation of linear regressions reports the 95% confidence bands of the best-fit line.

The mean FEV_1_ at baseline was 1,376 ± 154 mL in EOS− group and 1,553 ± 136 mL in EOS+ group. The changes from baseline (0 to 4 years) were −30 ± 43 mL in EOS− group and −114 ± 32 mL in EOS+ group, with a slope that was −12.2 ± 20.4 and −31.4 ± 13.6, respectively (Figure 1). Only in EOS+ group the slope was significantly different from zero (*P* < 0.05).

**Figure 2:**
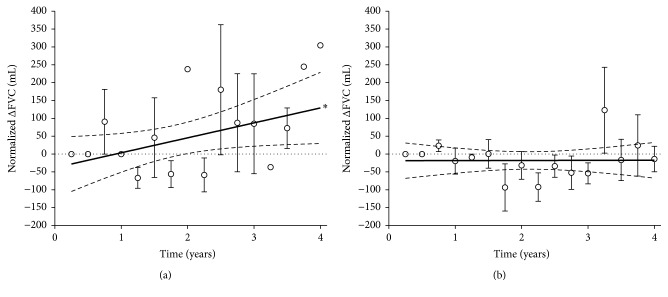
Normalized change from baseline in FVC in EOS− (a) and EOS+ (b) COPD patients; ^*∗*^slope significantly different from zero (*P* < 0.05). Data points expressed as mean ± SEM and the graphical representation of linear regressions reports the 95% confidence bands of the best-fit line.

Also the initial mean value of FVC in EOS− subjects (2,255 ± 202 mL) was lower than that in EOS+ subjects (2,602 ± 163 mL). The changes from baseline (0 to 4 years) were +129 ± 42 mL in EOS− group and −17 ± 27 mL in EOS+ group, with a slope that was 41.9 ± 20.2 and −0.3 ± 11.6, respectively (Figure 2). The slope was significantly different from zero (*P* < 0.05) only in EOS− group.

**Figure 3:**
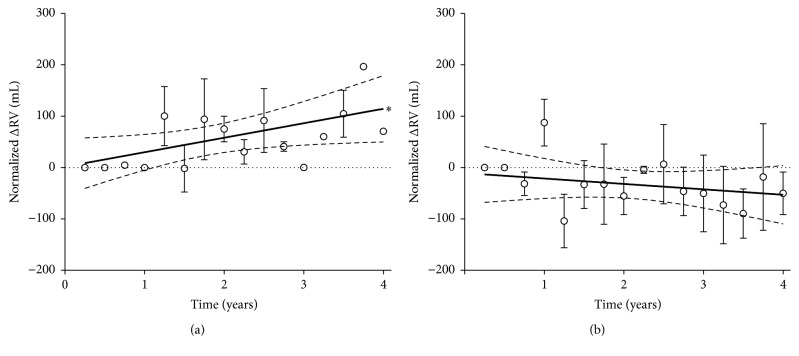
Normalized change from baseline in RV in EOS− (a) and EOS+ (b) COPD patients; ^*∗*^slope significantly different from zero (*P* < 0.05). Data points expressed as mean ± SEM and the graphical representation of linear regressions reports the 95% confidence bands of the best-fit line.

The initial mean value of RV in EOS− patients was 3,305 ± 322 mL compared with 3,435 ± 318 mL in EOS+ group. The changes from baseline (0 to 4 years) were +140 ± 27 mL in EOS− group and −32 ± 30 mL in EOS+ group, with a slope that was 28.2 ± 13.0 and −10.6 ± 13.0, respectively (Figure 3). The slope in EOS− group was significantly different from zero (*P* < 0.05).

**Figure 4:**
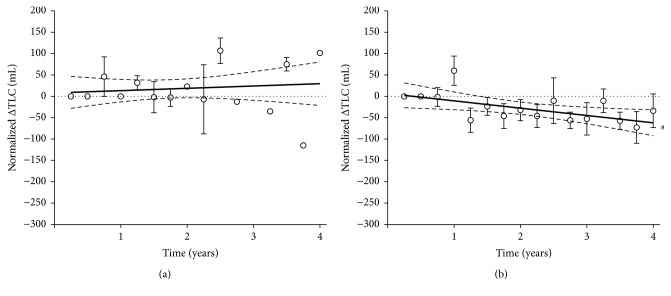
Normalized change from baseline in TLC in EOS− (a) and EOS+ (b) COPD patients; ^*∗*^slope significantly different from zero (*P* < 0.05). Data points expressed as mean ± SEM and the graphical representation of linear regressions reports the 95% confidence bands of the best-fit line.

At baseline, the mean value of TLC was 5,655 ± 362 mL in EOS− group and 6,140 ± 360 mL in EOS+ patients. The changes from baseline (0 to 4 years) were +30 ± 21 mL in EOS− group and −62 ± 16 mL in EOS+ group, with a slope that was 5.4 ± 10.1 and −17.1 ± 6.9, respectively (Figure 4). The slope in EOS+ group was significantly different from zero (*P* < 0.05).

## 4. Discussion

The results of this study suggest the presence of different populations within the COPD diagnostic category, based on the different changes over time of the spirometric values in function of the eosinophil blood counts. These data reinforce the hypothesis that COPD is a “disorder” rather than a disease, with different pathophysiological conditions that often converge clinically. This feature makes the nosological allocation of patients suffering from chronic and progressive respiratory obstruction complex.

In particular, the integrated evaluation of our data suggests that the different level of blood eosinophils in the two groups may have influenced independently the time course of the pulmonary function tests and identify two different clusters of subjects with specific disease characteristics or clinical phenotypes, the hyperinflator and the rapid decliner, respectively. A clinical phenotype is defined as “a single or combination of disease attributes that describe differences between individuals with COPD as they relate to clinically meaningful outcomes (symptoms, exacerbations, response to therapy, rate of disease progression, or death)” [17]. It is important to group patients in phenotypes because subjects included in the same subgroup/phenotype are expected to have similar disease, progression of disease, and response to treatments [18, 19].

We must admit that, regardless of our results, the association between eosinophilic inflammation and lung function decline is uncertain. In the past, Stănescu and colleagues [20] reported that increased numbers of eosinophils could be detected in the sputum of those with more severe airways obstruction. A negative correlation between FEV_1_ and the ratio of activated eosinophils to total eosinophils in endobronchial biopsies from subjects with COPD was demonstrated [21], and a similar negative correlation between FEV_1_ and sputum eosinophils and eosinophil cationic protein (ECP) was found [2]. In the study of Donaldson and colleagues [22], sputum eosinophils were related to FEV_1_ decline with allowance for covariates but not in the simpler analysis, which suggested a weak or confounded effect. Some years ago, D'Armiento and colleagues [14] described the association between level in lung lavage of eotaxin-1, a CC chemokine (CCL11) that binds to CC chemokine receptor 3 (CCR3) on the surface of eosinophils thereby inducing eosinophil activation and migration, and rapidly progressive disease in COPD patients with emphysema.

The Hokkaido COPD Cohort Study Group Investigators [15] reported that COPD patients, who displayed increased levels of circulating eosinophils, had lower levels of emphysema, more frequent chronic bronchitis symptoms, and slightly but significantly greater BMI and mainly maintained FEV_1_ over a period of 5 years. On the contrary, emphysema severity was independently associated with a rapid annual decline in FEV_1_ and higher circulating neutrophil count was also a significant predictor for the rapid decliners. However, in our population, EOS− patients had a significantly slow decline in FEV_1_ and larger increase in FVC and RV when compared with EOS+ subjects.

In the Evaluation of COPD Longitudinally to Identify Predictive Surrogate End-points (ECLIPSE) study, COPD subjects with blood eosinophil counts persistently ≥2% were slightly older and had a greater proportion of males and fewer current smokers than the other COPD groups [22]. They were also characterised by a higher FEV_1_% predicted, but the difference between groups in FEV_1_% predicted was small (about 3%) and, therefore, of debatable clinical relevance. This is in contrast to what we have observed in our population in which increased levels of circulating eosinophils identify patients with a faster decline in FEV_1_.

Recently, Lai and colleagues [23] suggested that persistence of eosinophilic inflammation invariably does not lead to rapid lung function decline and that this perhaps requires other features of susceptibility to disease progression, such as concomitant features of airway remodeling and changes in airway morphometry. Eltboli and colleagues [24] reported that there is a strong correlation between peripheral blood and bronchial eosinophils and reticular basement membrane thickening and suggested that the peripheral blood eosinophil count does identify COPD subjects with a greater tissue eosinophilia. A recent study documented that airway wall thickness is independently associated with a stronger decline in lung function [25]. On the basis of our results, we completely agree with Brightling and George [26] that it is too simplistic to consider the eosinophil as the crucial element leading the development of airflow obstruction, but it might play a role given the right environment and opportunity. In effect, there is documentation that a preferential distribution of eosinophils towards the airway lumen (i.e., low eosinophil numbers on biopsy with a high percentage of eosinophils in sputum) characterises patients with COPD with symptoms of chronic bronchitis compared with those without these symptoms [27]. Unfortunately, our data do not allow us to differentiate the EOS+ patients according to the presence or absence of symptoms of chronic bronchitis.

In the ECLIPSE study, a greater progression of emphysema was observed in patients with persistent lower blood eosinophil cell counts [28], and this finding fully confirms what we have observed, but, as already mentioned, the study published by the Hokkaido COPD Cohort Study Group Investigators [15] documented a negative correlation between levels of circulating eosinophils and levels of emphysema. Whatever the case may be, in the Subpopulations and Intermediate Outcomes in COPD Study (SPIROMICS), subjects with lower baseline eosinophil count (<1%) had more severe COPD, evidenced by a greater degree of obstruction, shorter 6-minute walk distances, and higher numbers of exacerbations, and were more commonly GOLD stage III or IV [29]. The possibility that there may be a greater progression of emphysema when the eosinophil count is <2% could be explained, according to Singh and colleagues [28], with the fact that a smaller number of eosinophils can lead to the presence of a greater number of other immune cell types such as neutrophils, which are known to cause emphysema. In any case, since our data were only functional, we cannot affirm that in our EOS− patients there was a progression of emphysema, but certainly there was an increase in lung hyperinflation. The lack of chest CT scan of these patients does not permit us to confirm the emphysema progression and further studies are needed.

In the EOS+ group there was a higher use of corticosteroids. Although the percent of peripheral eosinophils was not the criteria for choosing the therapy, we could not exclude that clinicians could have been influenced by that in their prescriptions.

*This Study Has Some Limitations.* The criterion to select only patients with stable COPD eosinophilia over time has significantly reduced the initial sample and the high number of patients excluded may have created bias in the population studied.

Taking into account the differences in the literature about the impact of eosinophils on COPD, we believe that our findings suggest that eosinophils are an important component of the clinical and functional manifestations in some patients with COPD, and the measure of their level in blood count is a useful tool in the nosological allocation of these patients, considering also that this parameter is easily measured. However, prospective studies on large populations of COPD patients are now needed in order to ascertain the real role of blood eosinophil count in influencing the pulmonary functional decline and the emphysema progression. Consequently, these studies should also assess the time course of other spirometric variables (including inspiratory capacity [IC] and functional residual capacity [FRC]), evaluate the efficiency of gas exchanges (diffusing capacity for carbon monoxide [DL_CO_] and CO transfer coefficient [*K*_CO_]), and carry out computed tomography to evaluate the relationship with COPD phenotypes and CT lung density changes. Such an approach, although complicated and expensive, would improve our understanding of COPD and help us in identifying effective personalised therapeutic strategies.

## Figures and Tables

**Table 1:** Demographic characteristics of the patients included in the study.

	EOS+ group (*n* = 23)	EOS− group (*n* = 12)	*P* value
Blood eosinophil count %, mean of the period (SE)	3.6 (0.21)	1.2 (0.10)	<0.001
Male/female, *n* (%)	18/5 (78/22)	8/4 (67/33)	NS
Age, mean (SE)	75.0 (1.38)	73.8 (1.97)	NS
Current smokers, *n* (%)	7 (30)	2 (17)	NS
Ex-smokers, *n* (%)	16 (70)	10 (83)	NS
Pack-year history of cigarette smoking, mean (SE)	34.0 (4.25)	61.0 (9.51)	<0.05
BMI kg/m^2^, mean (SE)	26.6 (1.13)	29.9 (2.03)	NS

EOS−: eosinophil count <2%; EOS+: eosinophil count ≥2%; BMI: body mass index; SE: standard error; and NS: not significant (*P* ≥ 0.05).

**Table 2:** Changes from baseline (0–4 yrs) and slope in EOS+ and EOS− COPD patients.

	Changes from baseline		Slope	
	EOS−	EOS+	EOS−	EOS+
FEV_1_ (mL)	−30 ± 43	−114 ± 32	−12.2 ± 20.4	−31.4 ± 13.6^*∗*^
FVC (mL)	+129 ± 42	−17 ± 27	41.9 ± 20.2^*∗*^	−0.3 ± 11.6
FEV_1_/FVC (%)	−6.2 ± 2.0	−3.4 ± 1.3	−2.3 ± 1.0^*∗*^	−0.9 ± 0.6
RV (mL)	+140 ± 27	−32 ± 30	28.2 ± 13.0^*∗*^	−10.6 ± 13.05
TLC (mL)	+30 ± 21	−62 ± 16	5.4 ± 10.1	−17.1 ± 6.9^*∗*^

^*∗*^Slope significantly different from zero (*P* < 0.05).
